# Alpha-Tocopherol Significantly Improved Squalene Production Yield of *Aurantiochytrium* sp. TWZ-97 through Lowering ROS levels and Up-Regulating Key Genes of Central Carbon Metabolism Pathways

**DOI:** 10.3390/antiox12051034

**Published:** 2023-04-30

**Authors:** Memon Kashif Ali, Xiuping Liu, Jiaqian Li, Xingyu Zhu, Biswarup Sen, Guangyi Wang

**Affiliations:** 1 Center of Marine Environmental Ecology, School of Environmental Science and Engineering, Tianjin University, Tianjin 300072, China; 6117000031@tju.edu.cn (M.K.A.); liuxp880@tju.edu.cn (X.L.); lijiaqian@tju.edu.cn (J.L.); xingyu_zhu@tju.edu.cn (X.Z.); 2 Key Laboratory of Systems Bioengineering (Ministry of Education), Tianjin University, Tianjin 300072, China; 3 Qingdao Institute for Ocean Technology of Tianjin University Co., Ltd., Qingdao 266237, China; 4 Center for Biosafety Research and Strategy, Tianjin University, Tianjin 300072, China

**Keywords:** thraustochytrids, antioxidants, squalene, supplementation, transcriptomics, quantitative PCR

## Abstract

Media supplementation has proven to be an effective technique for improving byproduct yield during microbial fermentation. This study explored the impact of different concentrations of bioactive compounds, namely alpha-tocopherol, mannitol, melatonin, sesamol, ascorbic acid, and biotin, on the *Aurantiochytrium* sp. TWZ-97 culture. Our investigation revealed that alpha-tocopherol was the most effective compound in reducing the reactive oxygen species (ROS) burden, both directly and indirectly. Adding 0.7 g/L of alpha-tocopherol led to an 18% improvement in biomass, from 6.29 g/L to 7.42 g/L. Moreover, the squalene concentration increased from 129.8 mg/L to 240.2 mg/L, indicating an 85% improvement, while the squalene yield increased by 63.2%, from 19.82 mg/g to 32.4 mg/g. Additionally, our comparative transcriptomics analysis suggested that several genes involved in glycolysis, pentose phosphate pathway, TCA cycle, and MVA pathway were overexpressed following alpha-tocopherol supplementation. The alpha-tocopherol supplementation also lowered ROS levels by binding directly to ROS generated in the fermentation medium and indirectly by stimulating genes that encode antioxidative enzymes, thereby decreasing the ROS burden. Our findings suggest that alpha-tocopherol supplementation can be an effective method for improving squalene production in *Aurantiochytrium* sp. TWZ-97 culture.

## 1. Introduction

Squalene is a terpenoid hydrocarbon (C_30_H_50_) with broad applications in food, medicine, and the cosmetic industry because of its wide range of biological properties. It shows biological activities against microbes (e.g., bacteria and fungi) and viruses and has antioxidant, tumor-suppressing, immunity-enhancing [1,2,3], and cholesterol-lowering properties [4,5]. Humans still mainly rely on deep-sea shark liver oil for squalene since it contains 40–70% of squalene by its dry weight [5]. This leads to the theft of sharks, resulting in massive damage to the marine ecosystem. Other competitive sources, including animals, plants, and microbes, show limited potential due to challenges such as seasons, covered areas, and lower squalene yields [6,7,8]. Because of their low squalene concentration and competition with agricultural land, plant products are widely accepted not to be an alternative resource [5,9]. Interestingly, microbes remain an unexplored source for squalene because of their ease of cultivation and low operational cost [10].

As one group of Labyrinthulomycetes, thraustochytrids have been reported to have good stability toward fermentation processes and genetic manipulation [8]. The strains used for squalene production mainly belong to the genus *Aurantiochytrium*, including *Aurantiochytrium mangrovei* FB3 [11], *Aurantiochytrium* sp. BR-MP4-A1 [12], *Aurantiochytrium* sp. 18W-13a [13], and *Aurantiochytrium* sp. TWZ-97 [14]. Members of this genus are heterotrophic unicellular marine protists, closely related to heterokont algae, and well known for their elevated DHA, squalene, and carotenoid production [11,12,13,14]. They are ubiquitous in diverse marine environments such as mangroves and mud flats, feeding mainly on organic substrates present in marine ecosystems [15]. Previous reports on these strains focused on optimizing traditional culture conditions [11,12,13,14,15,16]. Research on how antioxidant addition to culture media impacts squalene and biomass production of thraustochytrids is relatively scarce [17]. Cellular respiration and metabolism during aerobic growth lead to the generation of reactive oxygen species (ROS), which damage the cell and reduce the accumulation of lipids, antioxidants, carotenoids, and terpenoids [2,18,19,20,21,22]. In addition, ROS can cause damage to cellular DNA, proteins, and cell organelles, leading to cell injury and death. These ultimately result in less biomass and squalene yield [23,24,25]. Research on how antioxidant addition to culture media impacts squalene and biomass production of thraustochytrids is relatively scarce [17].

So far, a handful of chemicals have been tested on microalgae and thraustochytrids for their antioxidative properties and potential to improve lipid and squalene yields by providing them exogenously. These include melatonin [26], sesame oil [22,27], butylated hydroxy anisole [26,28], ascorbic acid [18,27], flaxseed oil [20], mannitol [17,29], biotin [17], and butylhydroxytoluene [26]. The exogenous addition of antioxidants has been reported to improve saturated and polyunsaturated fatty acids [30]. Furthermore, mannitol [17] and ascorbic acid [18] supplementation have shown increased squalene production in thraustochytrids. Nevertheless, studies on the impact of exogenous chemicals on squalene yield and their mechanisms of action are limited for thraustochytrids.

This study investigated the effects of alpha-tocopherol, mannitol, sesamol, melatonin, ascorbic acid, and biotin on squalene fermentation using *Aurantiochytrium*. sp. TWZ-97. This study provides strategies for enhancing squalene production through antioxidant supplementation and insight into the transcriptional regulation of metabolic pathways.

## 2. Materials and Methods

### 2.1. Strain and Culture Condition

*Aurantiochytrium* sp. TWZ-97 was maintained at room temperature on agar plates containing the growth medium described in our previous study [14]. Seed culture was prepared by inoculating a single colony from the agar plate into a 100 mL Erlenmeyer flask with 50 mL of growth medium and incubating the flask at 28 °C in an orbital shaker set for 24 h at 170 rpm.

### 2.2. Batch Fermentation Experiments

Various supplements such as alpha-tocopherol (g/L: 0.5, 0.6, 0.7, 0.8), mannitol (g/L: 0.5, 1.0, 1.5), melatonin (mg/L: 0.25, 0.30, 0.35), sesamol (mg/L: 70, 87.5, 105), ascorbic acid (g/L: 3, 6, 9), and biotin (mg/L: 0.01, 0.05, 0.1) were individually added to the culture medium to evaluate their effects on growth and squalene production in shake flasks. These concentrations were selected based on previous studies [17,18,22,30,31]. The chemicals were purchased from Sigma-Aldrich (St. Louis, MO, USA). Alpha-tocopherol, sesamol, biotin, and melatonin were dissolved in DMSO, whereas mannitol and ascorbic acid were dissolved in water. The stock solutions of alpha-tocopherol (3.5 g/100 mL), mannitol (5 g/100 mL), melatonin (125 mg/L), sesamol (350 mg/100 mL), ascorbic acid (15 g/100 mL), biotin (100 mg/L), and others were kept in the dark at −20 °C. All stocks were sterilized by passing through a 0.25 µm membrane filter. Batch fermentation was conducted in a 100 mL Erlenmeyer flask containing 50 mL of production medium, as described in our previous study [17]. At 0 h of fermentation, the supplements were individually added.

To verify shake flask experimental results, batch fermentation was carried out in a 5 L bioreactor (Model: SY9000-V9, Shanghai Dong Ming Industrial Co., Ltd., Shanghai, China) equipped with DO and pH electrodes, a temperature sensor, an impeller, and an air pump. The working volume of the bioreactor was 2.5 L. Fermentation was carried out at 28 °C, 170 r/min for 72 h. At 0 h of fermentation, an appropriate volume of alpha-tocopherol stock was added to the culture to achieve a final concentration of 0.7 g/L.

### 2.3. Analytical Methods

The intracellular ROS levels and total antioxidant capacity (T-AOC) were measured according to the procedures described in our previous study [29]. The TAO-C was calculated by Ferric Reducing Ability of Plasma (FRAP) assay [30,31]. In this assay, the reduction of ferric to ferrous ions at low pH yields a colored ferrous-tripyridyltriazine complex using the T-AOC assay kit (Solarbio, Beijing, China). In brief, microbial cells were collected every 12 h interval and pelleted by centrifuging at 4 °C, 4000 rpm for 5 min. The collected pellet was washed briefly with deionized water. The resulting cell pellet was transferred into a mortar and crushed in liquid nitrogen with a pestle. The resulting cell powder was suspended in the extraction buffer supplied in the kit. Then, the suspension solution was centrifuged at 4 °C, 10,000 rpm for 10 min. The supernatant was mixed with three reagent solutions (7:1:1) provided in the kit. The absorbance of the reaction solution was recorded at 593 nm, and the total antioxidant capacity (U/mL) was calculated by following the manufacturer’s instructions. Cellular ROS were detected using the Reactive Oxygen Species Assay Kit (Meilun, Shenzhen, China), which contains DCFH-DA (2,7-Dichlorodi-hydrofluorescein diacetate) [32] a non-fluorescence dye to pass over the cell membrane. This probe does not disrupt the cell layers and simply labels the ROS with an illumination. The cells were collected every 12 h and washed with distilled water; the DI-water-washed cells initially were treated with DTT (Sigma-Aldrich, St. Louis, MO, USA) snailase (Solarbio, Beijing, China) to soften cell wall and then incubated with DCFH-DA diluted to 10 μM with 10 mM PBS buffer and were directly treated with the dye and incubated at 37 °C for 40 min in dark. After removing extra dye in the reaction with 10 mM PBS, the excitation wavelength was carried out at 488 nm and emission at 525 nm at 450 V gain on fluorescence spectrophotometer F97 Pro (Lengguang, Shanghai, China).

Residual glucose levels were estimated following the methods mentioned in our previous study [17]. Briefly, 1 mL fermentation broth was centrifuged for 10 min at 10,000 rpm and 4 °C. The supernatant was transferred and diluted to 10× with distilled water in a new tube for glucose concentration analysis using the Glu Kit (Biosino Bio-Technology and Science inc., Beijing, China). The intensity of the red-colored products from the kit assay was recorded at the wavelength of 505 nm using the spectrophotometer manufactured by (Multiskan GO, Thermo Scientific, Waltham, MA, USA).

The dry cell weight (DCW) and squalene concentration were quantified according to the methods described elsewhere [14,33].

### 2.4. RNA Sequencing and Bioinformatics Analysis

To probe the effect of alpha-tocopherol on the transcriptional regulation of squalene biosynthesis, the transcriptome of TWZ-97 strain was analyzed with (test) and without (control) the supplementation. Triplicate culture samples from the control and test groups were collected at 42 h of fermentation for RNA sequencing (RNA-Seq). Each sample was centrifuged at 12,000 rpm for 5 min at 4 °C; the pellets were frozen directly in liquid nitrogen and then stored at −80 °C.

Poly(A) RNA sequencing library of each sample was prepared following Illumina’s TruSeq-stranded-mRNA sample preparation protocol. RNA integrity was checked with Agilent Technologies 2100 Bioanalyzer. Poly(A)-tail-containing mRNAs were purified using oligo-(dT) magnetic beads with two rounds of purification. After purification, poly(A) RNA was fragmented using a divalent cation buffer at elevated temperature. Quality control analysis and quantification of the sequencing library were performed using Agilent Technologies 2100 Bioanalyzer High Sensitivity DNA Chip. Paired-ended sequencing was performed on Illumina’s NovaSeq 6000 sequencing system. Cutadapt [34] and in-house Perl scripts were used to remove the reads containing adaptor contamination, low-quality, and undetermined bases. The sequence quality was verified using FastQC (http://www.bioinformatics.babraham.ac.uk/projects/fastqc/ (accessed on 1 January 2021), including the Q20, Q30 and GC-content of the clean data. All downstream analyses were based on clean data of high quality. De novo assembly of the transcriptome was performed with Trinity 2.4.0 [35]. All assembled unigenes were aligned against the non-redundant (Nr) protein database (http://www.ncbi.nlm.nih.gov/ (accessed on 1 January 2021)), Gene ontology (GO) (http://www.geneontology.org (accessed on 1 January 2021)), SwissProt (http://www.expasy.ch/sprot/ (accessed on 1 January 2021)), Kyoto Encyclopedia of Genes and Genomes (KEGG) (http://www.kegg.jp/kegg/ (accessed on 1 January 2021)) and eggNOG (http://eggnogdb.embl.de/ (accessed on 1 January 2021)) databases using DIAMOND [36] with a threshold of E-value < 0.00001. Library construction, transcriptome sequencing, and bioinformatics analysis were conducted at LC Sciences (Houston, TX, USA).

Salmon [37] was used to quantify the expression of transcripts/unigenes by calculating TPM [38]. This efficient tool was used to calculate transcript expressions in RNA-seq data providing accurate and fast results by removing fragment wise GC content bias. It links modern double-phase models, i.e., parallel inference algorithm and feature-rich bias. The differentially expressed unigenes were selected with log2 (fold change) > 1 or log2 (fold change) < −1 and with statistical significance (*p* value < 0.05) by R package edgeR [39].

### 2.5. Quantitative PCR

The total RNA was extracted from control and test (supplemented with alpha-tocopherol) samples using E.Z.N.A. plant RNA kit (Omega Bio-tek, Inc., Norcross, GA, USA). cDNA was synthesized using random primers with SPARKscript 1st Strand cDNA Synthesis Kit (with gDNA Eraser) (SparkJade, China). Gene-specific primers were designed for glucose-6-phosphate isomerase, squalene synthase, and glucose-6-phosphate dehydrogenase (reference gene) (Table 1). To confirm the primers and cDNA, PCR was conducted in a 25 μL reaction volume, containing 12.5 μL 2X Taq pol PCR master mix, 1μL of each primer (10 μM), 1 μL cDNA, and 9.5 μL nuclease-free water. The PCR program was set to 95 °C for 3 min, 34 cycles of 95 °C for 30 s, 55 °C (59 °C for SQS) for 30 s, 73 °C for 30 s, and 10 min for final elongation. The size of the PCR product was checked by 2% agarose gel electrophoresis.

Quantitative PCR (qPCR) assays were performed in triplicate on a CFX Connect™ Real-Time System (Bio-Rad Laboratories, Inc., Hercules, CA, USA) with ChamQ™ SYBR qPCR Master Mix (Vazyme, Nanjing, China). QPCR was performed in a 10 μL reaction volume, containing 5 μL qPCR master mix, 0.3 μL of each primer (10 μM), 0.8 μL cDNA, and 3.6 μL nuclease-free water. The PCR program was set to 95 °C for 3 min, followed by 39 cycles of 95 °C for 10 s, 55 °C (59 °C for SQS) for 30 s, and then 72 °C for 20 s. The expression levels of genes (SQS and GPI) with reference to the G6PDH gene were calculated following the 2^−ΔΔCT^ method [39]. The melt curve analysis showed a single peak for each gene.

### 2.6. Statistical Analysis

The data are expressed as a mean ± standard deviation (SD). The significance test (one-way ANOVA) was performed in Origin Pro software (student version).

## 3. Results and Discussion

### 3.1. Effect of Supplementation on Squalene Fermentation

This study evaluated various bioactive compounds for their effects on squalene fermentation by the TWZ-97 strain. The results showed that melatonin (at concentrations of 0.25 g/L and 0.30 g/L), sesamol (at concentrations of 87.5 mg/L and 105 mg/L), ascorbic acid (at a concentration of 9 g/L), and biotin (at concentrations of 0.01, 0.05, and 0.10 mg/L) all had a significant positive impact on the biomass of the TWZ-97 strain (Table 2). However, in the case of squalene production, our study found that alpha-tocopherol (at concentrations of 0.5 to 0.8 g/L), mannitol (at a concentration of 1.0 g/L), sesamol (at concentrations of 87.5 g/L and 105 g/L), and ascorbic acid (at concentrations of 6 g/L and 9 g/L) all had a significant positive impact. Among these supplements, alpha-tocopherol at a concentration of 0.7 g/L was the most effective, with the highest squalene production (170.36 ± 1.7 mg/L) and yield (27.2 ± 2.8 mg/g). This resulted in an increase of 31.2% in squalene production and 37.8% in yield.

**Figure 1:**
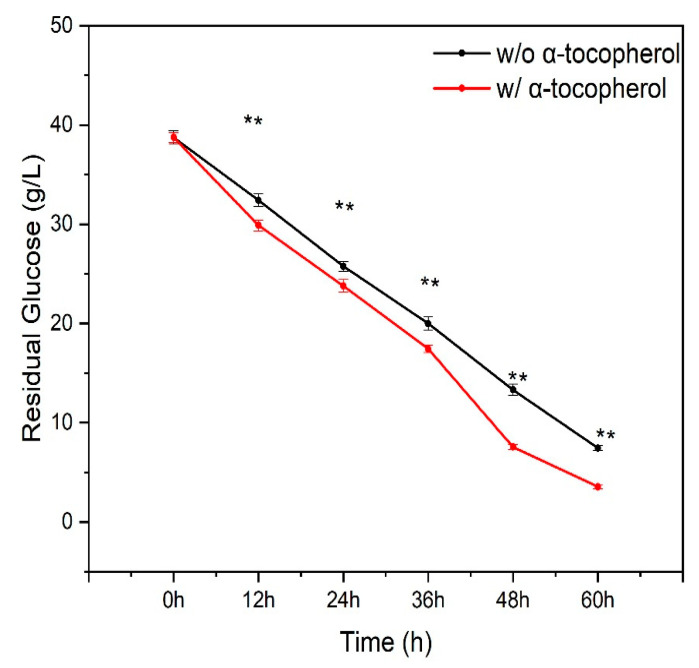
Comparison of glucose consumption patterns between with and without alpha-tocopherol-supplemented groups during fermentation by TWZ-97 strain. The significance code '**' indicated a significant difference at (*p*-value < 0.01) between the supplemented and non-supplemented ROS data.

The results of this study revealed that the residual glucose content of the alpha-tocopherol-supplemented culture was significantly lower during the fermentation period (i.e., 12 h–60 h) when compared to the culture without alpha-tocopherol supplementation (Figure 1). This observation indicated that adding alpha-tocopherol improved glucose uptake in the TWZ-97 strain. Similar effects have been reported with flaxseed oil supplementation [20] and the addition of ascorbic acid [18]. These findings suggest that media supplementation with bioactive compounds can improve biomass and squalene production by increasing glucose uptake into the cells.

We performed a 5 L batch fermentation experiment to evaluate the effectiveness of supplementation using 0.7 g/L alpha-tocopherol. Our results showed that the squalene concentration and yield reached 240.3 ± 0.9 mg/L and 32.5 ± 2.0 mg/g, respectively, which were 41.1% and 19.5% higher than the results (170.3 mg/L and 27.21 mg/g) obtained from the 100 mL flask culture and 27.4% and 72% higher than previously reported values of 188.6 mg/L and 18.83 mg/g for this strain. In addition, the biomass increased from 6.29 g/L to 7.42 g/L, which was 18% higher compared to the results from the 100 mL flask culture. These results supported the efficacy of alpha-tocopherol in improving squalene and biomass production. More importantly, this study provides the first evidence that alpha-tocopherol supplementation can increase biomass and squalene yield in thraustochytrids.

### 3.2. Effect of Alpha-Tocopherol on Intracellular ROS Level and T-AOC

**Figure 2:**
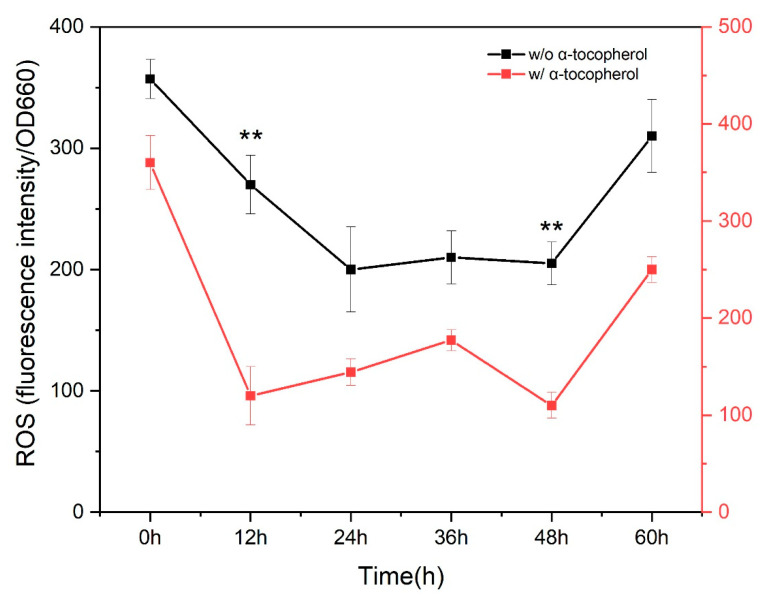
Time course profile of intracellular reactive oxygen species (ROS) levels in non-supplemented and supplemented cultures of TWZ-97 strain. The significance code '**' indicated a significant difference at (*p*-value < 0.01) between the supplemented and non-supplemented ROS data.

To further understand the biological effects on the TWZ-97 strain, we investigated the antioxidant properties of alpha-tocopherol by comparing the levels of intracellular ROS and T-AOC in control and supplemented TWZ-97 cultures. The results showed that alpha-tocopherol supplementation lowered ROS levels throughout fermentation (Figure 2). These findings suggest that alpha-tocopherol can effectively protect TWZ-97 cells from oxidative damage caused by ROS during fermentation. The high ROS levels at the start of fermentation can be attributed to the seed culture, as reported in previous studies [40,41]. Furthermore, the lowest ROS level was detected at 48 h of fermentation in both groups, possibly due to the intracellular accumulation of squalene and carotenoids, as explained in some previous studies [18,40,41]. After 48 h of fermentation, the ROS level increased in both the non-supplemented and supplemented cultures. The lower ROS levels throughout the fermentation in the supplemented group can be attributed to alpha-tocopherol’s indirect and direct antioxidative effects on the TWZ-97 strain. The defense system against oxidative stress exists in two different mechanisms: direct and indirect antioxidant effects [19,42]. In the direct antioxidant effect, the ROS are directly adsorbed to the antioxidants and detoxified, whereas in the indirect method, the expressions of genes for antioxidant enzymes (such as superoxide dismutase (SOD) and catalase (CAT)) are involved to reduce the oxidative burden.

**Figure 3:**
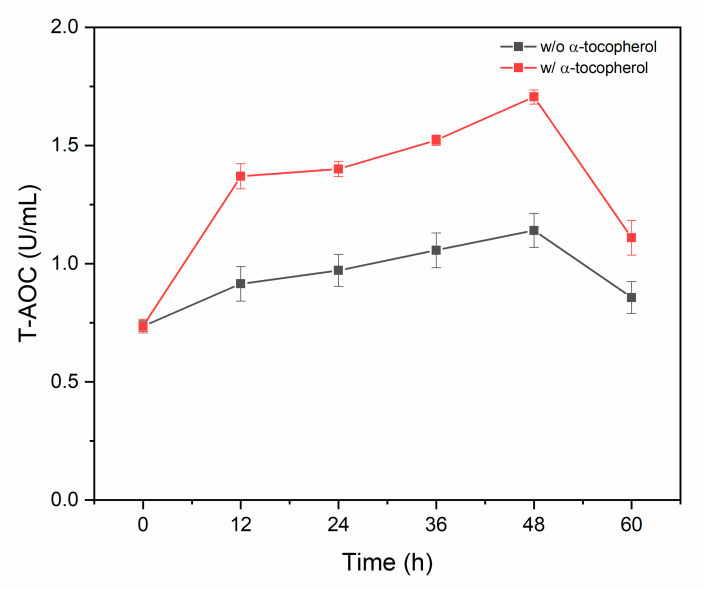
Time course profile of total antioxidant capacity (T-AOC) levels in non-supplemented and supplemented cultures of TWZ-97 strain.

Our study examined the impact of alpha-tocopherol supplementation on the T-AOC of TWZ-97 culture throughout the fermentation process (Figure 3). The results revealed that while T-AOC initially remained low in non-supplemented and supplemented cultures, it increased during the 12 h and 48 h fermentation periods. However, a decline in T-AOC was observed after 48 h in both cultures. This decline in T-AOC may be linked to the high levels of ROS produced during fermentation. Further research is needed to understand the underlying mechanisms behind this decline in T-AOC.

Some research has found that alpha-tocopherol can promote the growth of certain microorganisms, such as lactic acid bacteria [43]. This biological activity is likely due to its antioxidant properties, which can help protect the microbe from the harmful effects of ROS. However, in other studies, researchers have found that alpha-tocopherol can inhibit the growth of certain microorganisms, such as pathogenic bacteria [44], because alpha-tocopherol can disrupt the membrane structure of the microorganism, thus making it difficult for them to survive. It should be noted that the effect of alpha-tocopherol on microorganisms can vary depending on the species, study conditions, and alpha-tocopherol concentration. More research is needed to fully understand the effects of alpha-tocopherol on different microorganisms.

### 3.3. Transcriptional Regulation of Metabolism

Alpha-tocopherol supplementation impacted multiple metabolic pathways significantly compared to the control group. Our analysis revealed that a total of 3557 genes were significantly overexpressed (FDA ≤ 0.05), and 1001 genes were down-regulated (Table 3). Our findings indicate that the genes predominantly involved in pathways such as glycolysis, gluconeogenesis, the pentose phosphate pathway (PPP), the fructose mannose pathway, the tricarboxylic acid (TCA) cycle, and free radical exchange pathways were among those overexpressed (Table 4, Figures S1 and S2). The genes encoding key enzymes in the gluconeogenesis pathway, including hexokinase, glucose 6-phosphate isomerase (GPI), 6-phosphate fructokinase, fructose 1,6-bisphosphate, triose phosphate isomerase, phosphoglycerate kinase, enolase, and pyruvate carboxylase, were significantly overexpressed in glycolysis. The overexpression of these genes suggested increased carbon flow in the cell, as described in a previous study [45]. Moreover, it has been reported that increased acetyl CoA production can boost squalene production [46]. 

We found that PPP, an NADPH generation pathway linked to glycolysis at the initial stage, was also overexpressed (Table 4). It has been reported that elevated NADPH production can increase squalene production [47]. NADPH acts as a cofactor for the key enzyme SQS in the mevalonate pathway [48]. In PPP, the overexpressed genes encode 6-phosphogluconate dehydrogenase, 6-phosphogluconolactonase, trans-aldolase, fructose 1,6-bisphosphatase I, 6-phosphofructokinase, transketolase, fructose 6-bisphosphate aldolase, and ribose-phosphate pyrophosphokinase. These enzymes can result in an elevated NADPH [49] and an enhancement in squalene production [50,51].

Alongside glycolysis and PPP, the galactose metabolism pathway was also overexpressed. Genes encoding enzymes, such as UTP-glucose-1-phosphate uridyl-transferase, UDP-glucose 4-epimerase, hexokinase, alpha-galactosidase, and maltase-glucoamylase, were overexpressed (Table 4). These enzymes regenerate glucose, fructose, and galactose molecules. Therefore, galactose was likely recycled in the process to provide a continuous supply, while glucose entered the glycolysis pathway and fructose was metabolized in the fructose mannose pathway (FMP). In FMP, the overexpressed genes encoding enzymes that included hexokinase, mannose-6-phosphate isomerase, phospho-mannomutase, fructose 1,6-bisphosphatase I, 6-phosphofructokinase, GDP mannose 4,6-dehydratase, and GDP-L-fucose synthase. Overall, our results suggest that the significant enrichment of the central metabolic pathways (Figure S2) resulted in an ample flow of energy to the TCA, the optimal consumption of glucose in the cell, and the production of a substantial amount of NADPH.

The formation of acetyl CoA is a crucial step that fuels the TCA cycle and provides the necessary building units for the biosynthesis of fatty acids and isoprenoids [52,53]. In the present study, genes encoding enzymes involved in the TCA cycle, such as citrate synthase, isocitrate dehydrogenase, aconitate hydratase, succinyl CoA synthetase alpha subunit, succinate dehydrogenate, fumarate hydratase, and malate dehydrogenase, were significantly overexpressed. Furthermore, the interconversion step of acetaldehyde and alcohol by alcohol dehydrogenase was also overexpressed (Table 4). The overexpression of these genes possibly fueled the energy generation inside cells, which enhanced the energy flow towards the mevalonate (MVA) pathway, resulting in a significant increase in squalene production. In the MVA pathway, the essential genes involved in squalene biosynthesis, including acetoacetyl CoA synthetase, hydroxymethylglutaryl CoA synthase, and farnesyl-diphosphate farnesyltransferase (SQS), were overexpressed. In contrast, the gene encoding for the enzyme responsible for converting squalene to sterol, sterol 1–4 alpha-demethylase, was significantly down-regulated. These findings provide the mechanisms for the increased production of squalene in the supplemented culture.

### 3.4. Transcriptional Regulation of Antioxidative Pathways

Alpha-tocopherol has been shown to be a potent antioxidant, and thus, the genes related to pathways involved in scavenging ROS were analyzed in this study. The analysis revealed that genes involved in ROS scavenging, such as superoxide dismutase, catalase, gamma-glutamyl cysteine synthase, and glutathione peroxidase, were significantly overexpressed (Table 4, Figure S3). These results indicated that reduced ROS levels and increased T-AOC can enhance biomass and squalene production.

The results obtained through the transcriptomic analysis were further validated using the qPCR method. The reference gene, G6PDH, was used as a normalizer, and the expression levels of SQS and GPI were quantified. The results revealed that SQS and GPI genes showed 5.49- and 3.89-fold higher expression levels in the supplemented culture, respectively, compared to the non-supplemented culture.

## 4. Conclusions

The addition of alpha-tocopherol to the culture of *Aurantiochytrium* sp. TWZ-97 had significant positive effects on both its growth and squalene production. Adding 0.7 g/L of alpha-tocopherol led to a reduction in the burden of ROS and an improvement in biomass yield and squalene content. These effects were mediated by the overexpression of genes involved in glucose uptake, including those related to glycolysis, the PPP, the galactose pathway, the fructose–mannose pathway, and the TCA cycle. The higher energy flow resulting from the overexpression of these central metabolic pathways possibly led to the upregulation of genes involved in squalene biosyntheses, such as HMG-CoA, acetoacetyl CoA synthetase, and SQS. These findings suggest that adding alpha-tocopherol could be a valuable strategy for increasing thraustochytrids’ biomass yield and squalene content in various biotechnological applications.

## Figures and Tables

**Table 1:** Details of primers used in PCR and qPCR experiments.

Gene	Accession Number	Primer	T_m_	Reference
Glucose 6-phosphate dehydrogenase	EC:1.1.1.44	F 5′GCTATGCCGTCTCCGTCTT′3 R 3′ACCTCTGTAGTTCCTCCTGCTA′5	55°	This study
Glucose 6-phosphate isomerase	EC:5.3.1.1	F 5′CCATCACggACATCATCAACAT’3 R 3′TGAAGGTCTTGGAGGCGATTA′5	55°	This study
Squalene synthase	EC:2.5.1.21	F 5′ACGGCACAGATGACGCTAA′3 R 3′TCAACAAGGTCCTCAAGGTAGT′5	59°	This study

**Table 2:** Comparative effects of different supplements on the biomass, squalene concentration, and squalene yield of *Aurantiochytrium* sp. TWZ-97 strain.

Supplement Name	Supplement Concentration	Biomass (g/L)	Squalene (mg/L)	Yield (mg/g)
Water		7.64 ± 0.32	139.05 ± 1.03	18.21 ± 0.67
DMSO		6.54 ± 0.29	129.79 ± 10.52	19.82 ± 1.26
alpha-tocopherol (g/L)	0.50	7.02 ± 0.50	159.8 ± 1.80 *	22.83 ± 1.50
	0.60	6.57 ± 0.61	163.21 ± 1.50 *	24.96 ± 2.38 *
	0.70	6.29 ± 0.60	170.36 ± 1.70 **	27.22 ± 2.84 *
	0.80	7.04 ± 0.14	163.43 ± 1.41 *	23.21 ± 0.33 *
Mannitol (g/L)	0.50	7.16 ± 0.40	140.21 ± 2.41	19.6 ± 0.77
	1.0	7.22 ± 0.24	142.93 ± 2.04 *	19.81 ± 0.56 *
	1.5	7.72 ± 0.28	140.89 ± 2.60	18.26 ± 0.96
Melatonin (mg/L)	0.25	8.08 ± 0.62 *	140.8 ± 0.64	17.47 ± 1.27
	0.30	8.19 ± 0.30 **	143.02 ± 0.77	17.46 ± 0.64 *
	0.35	7.19 ± 0.43	136.55 ± 1.57	19.02 ± 0.94
Sesamol (mg/L)	70.0	8.18 ± 0.44	139.07 ± 1.55	17.11 ± 1.76
	87.5	7.95 ± 0.15 **	78.61 ± 1.69 **	9.88 ± 0.16 **
	105.0	7.86 ± 0.15 **	79.14 ± 1.12 **	10.06 ± 0.05 **
Ascorbic acid (g/L)	3.0	8.091 ± 0.54	139.03 ± 0.93	17.27 ± 1.49
	6.0	7.61 ± 0.02	130.28 ± 1.82 **	17.1 ± 0.21 **
	9.0	6.61 ± 0.03 **	99.75 ± 1.95 **	15.08 ± 0.26 *
Biotin (mg/L)	0.01	8.47 ± 0.41 **	140.93 ± 0.69	16.66 ± 0.74 *
	0.05	8.54 ± 0.61 *	145.08 ± 1.60	17.02 ± 1.06 *
	0.10	8.14 ± 0.09 **	141.19 ± 0.72	17.34 ± 0.20 *

Note: The data represent the mean ± SD of triplicate samples collected at 60 h of cultivation. The statistical significance between the control group (w/o supplement) and test group (w/supplement) is indicated by ‘*’ (*p* ≤ 0.05) or ‘**’ (*p* ≤ 0.01).

**Table 3:** Summary of annotated and differentially expressed genes (DEGs).

	Number	Ratio (%)
Total DEGs	31441	100
Up-regulated DEGs (FDR ≤ 0.05)	3557	11.32
Down-regulated DEGS (FDR ≤ 0.05)	1001	4.2
GO annotated	11805	37.55
KEGG annotated	6249	19.88
Pfam annotated	10951	34.83
SwissProt annotated	11106	35.32
Eggnog	13071	41.57
NR annotated	8518	27.09

**Table 4:** Regulation of the key genes encoding enzymes in specific metabolic pathways.

Pathway	Annotation Platform	Enzyme	Gene/Transcript ID	Log_2_FC
Mevalonate	EC:2.3.3.10	Hydroxymethylglutaryl-CoA synthase	TRINITY_DN14298_c2_g8	8.65
	EC:1.1.1.34	3-hydroxy-3-methylglutaryl-CoA reductase	TRINITY_DN14778_c4_g8	4.43
	EC:1.14.13.70	Sterol 14-demethylase	TRINITY_DN15500_c0_g10	−1.16
	EC:2.5.1.21	Squalene synthase	TRINITY_DN17295_c0_g5	4.70
Galactose	EC:2.7.7.23/83	UTP-glucose-1-phosphate uridylyltransferase	TRINITY_DN15135_c0_g3	6.08
	GO:000382	Galactosidase	TRINITY_DN16025_c0_g4	7.83
	EC:5.1.3.2	UDP-glucose 4-epimerase	TRINITY_DN17242_c0_g2	10.23
	EC:3.2.1.20	Maltase-glucoamylase	TRINITY_DN12554_c0_g1	8.07
Glycolysis/Gluconeogenesis	EC:1.1.1.2	Alcohol dehydrogenase	TRINITY_DN14468_c0_g1	9.96
Glycolysis	EC:2.7.1.11	6-phosphofructokinase/Hexokinase	TRINITY_DN14626_c1_g7	3.58
	EC:6.4.1.1	Acetyl-CoA carboxylase	TRINITY_DN15131_c0_g1	1.29
	EC:5.3.1.1	Triosephosphate isomerase	TRINITY_DN16634_c0_g3	3.37
	EC:2.7.2.3	Phosphoglycerate kinase	TRINITY_DN16564_c0_g11	2.48
	EC:5.3.1.9	Glucose-6-phosphate isomerase 2	TRINITY_DN3634_c0_g1	3.03
	EC:6.4.1.1	Pyruvate carboxylase	TRINITY_DN15131_c0_g1	1.29
	EC:4.2.1.11	Enolase	TRINITY_DN12930_c0_g1	1.97
TCA cycle	EC:2.3.3.1	Citrate synthase	TRINITY_DN14908_c0_g11	4.12
	EC:4.2.1.3	Aconitate hydratase	TRINITY_DN15062_c1_g11	4.37
	EC:1.1.1.37	Malate dehydrogenase	TRINITY_DN15862_c1_g9	3.27
	EC:1.1.1.41	Isocitrate dehydrogenase	TRINITY_DN401_c0_g1	4.40
	EC:6.2.1.4/5	Succinyl-CoA synthetase	TRINITY_DN15910_c1_g2	6.04
	GO:0000104	Succinate dehydrogenase	TRINITY_DN15910_c1_g1	4.21
	EC:4.2.1.2	Fumarase	TRINITY_DN9827_c0_g1	4.45
Pentose phosphate (PPP)	EC:2.2.1.2	Transaldolase	TRINITY_DN10039_c0_g1	4.14
	EC:2.2.1.1	Transketolase	TRINITY_DN14437_c0_g5	3.68
PPP/Fructose-mannose	EC:3.1.3.11	Fructose-1,6-bisphosphatase	TRINITY_DN16377_c0_g4	9.24
	EC:2.7.1.11	6-Phosphofructokinase	TRINITY_DN16557_c1_g1	3.70
	EC:1.1.1.44/343	6-Phosphogluconate dehydrogenase	TRINITY_DN9634_c0_g2	3.91
	EC:3.1.1.31	6-Phosphogluconolactonase	TRINITY_DN18000_c0_g1	3.54
Antioxidative system	EC:1.11.1.6	Catalase	TRINITY_DN13811_c0_g1	1.09
	EC:6.3.2.3	Glutathione synthase	TRINITY_DN15025_c0_g14	6.18
	EC:1.11.1.9	Glutathione peroxidase	TRINITY_DN15473_c0_g19	4.09
	EC:6.3.2.3	Glutathione synthetase	TRINITY_DN16162_c1_g8	5.92
	EC:1.15.1.1	Superoxide dismutase	TRINITY_DN18100_c0_g1	3.38
Fructose-mannose	EC:5.3.1.8	Mannose-6-phosphate isomerase	RINITY_DN14626_c1_g1	6.70
	EC:4.2.1.47	GDP-mannose 4,6-dehydratase	TRINITY_DN14934_c1_g13	6.77
	EC:5.4.2.8	Phosphomannomutase	TRINITY_DN17527_c1_g5	6.27
	EC:1.1.1.271	GDP-L-fucose synthase	TRINITY_DN5650_c0_g1	6.15

## Data Availability

All of the data is contained within the article and the Supplementary Materials.

## Supplementary Materials

The following supporting information can be downloaded at: https://www.mdpi.com/article/10.3390/antiox12051034/s1, Figure S1: Detailed graphical representation of genes expressed in multiple induced pathways and involved in higher biomass, squalene production in supplemented sample. Glyceraldehyde 3-phosphate (G3P) phosphoglycerate kinase (PKG) Glyceraldehyde 3-phosphate (G3PDH), fructose bisphosphate aldolase (FBAL/ALD0); multiple red arrows show higher energy glow in system, and octagonal structure shows alpha-tocopherol.; Figure S2: KEGG enriched pathways between supplemented and non-supplemented groups; Figure S3: Graphical representation of ROS types, generation sites, damage sites and genes responsible for neutralization of ROS in biological system. Red box shows up-regulated genes and blue box shows down-regulated genes.
