# Supplementary material for: Alpha-Tocopherol Significantly Improved Squalene Production Yield of Aurantiochytrium sp. TWZ-97 through Lowering ROS levels and Up-Regulating Key Genes of Central Carbon Metabolism Pathways
Source: Antioxidants (Basel). 2023 Apr 30;12(5):1034. doi: 10.3390/antiox12051034 (PMC10215387; doi:10.3390/antiox12051034)
Supplement: Supplementary file 1 [file antioxidants-12-01034-s001.zip › antioxidants-2340835-supplementary.pdf]

Supplementary Information

Alpha-Tocopherol Significantly Improved Squalene Production Yield of *Aurantiochytrium* sp. TWZ-97 Through Lowering ROS levels and Up-regulating Key Genes of Central Carbon Metabolism Pathways

Memon Kashif Ali[1], Xiuping Liu[1], Jiaqian Li[1], Xingyu Zhu[1], Biswarup Sen[1, *], Guangyi Wang[1,2,3,4 *]

[1]Center of Marine Environmental Ecology, School of Environmental Science and Engineering, Tianjin University, Tianjin 300072, China

[2]Key Laboratory of Systems Bioengineering (Ministry of Education), Tianjin University, Tianjin 300072, China

[3]Qingdao Institute for Ocean Technology of Tianjin University Co., Ltd., Qingdao 266237 China

[4]Center for Biosafety Research and Strategy, Tianjin University, Tianjin 300072, China

***Corresponding author**

Email: bsen@tju.edu.cn (B.S); gywang@tju.edu.cn (G.W)

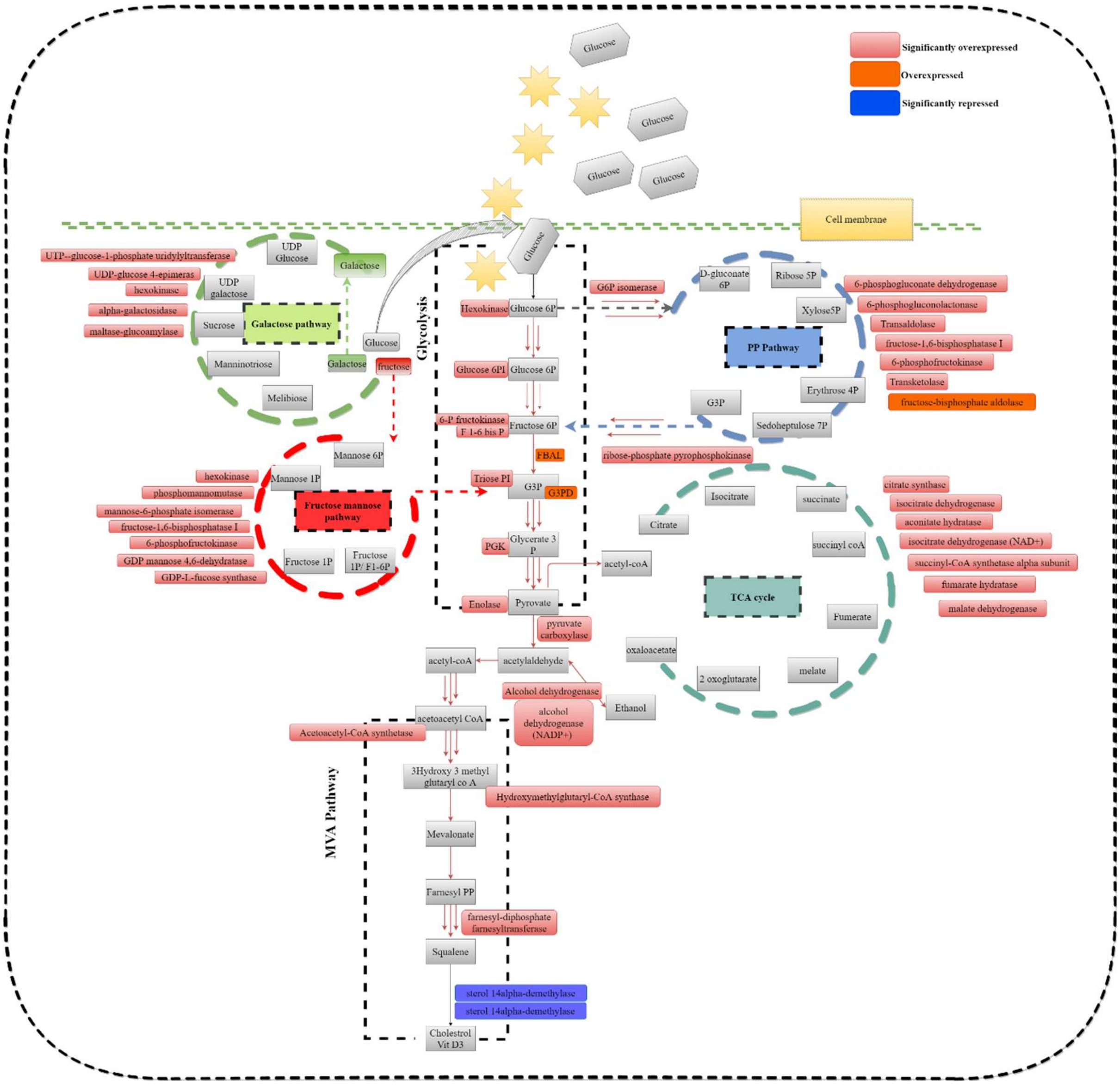

Figure S1. Detailed graphical representation of genes expressed in multiple induced pathways and involved in higher biomass, squalene production in supplemented sample. Glyceraldehyde 3-phosphate (G3P) phosphoglycerate kinase (PKG) Glyceraldehyde 3-phosphate (G3PDH), fructose bisphosphate aldolase (FBAL/ALD0), multiple red arrows show higher energy glow in system, octagonal structure shows alpha-tocopherol.

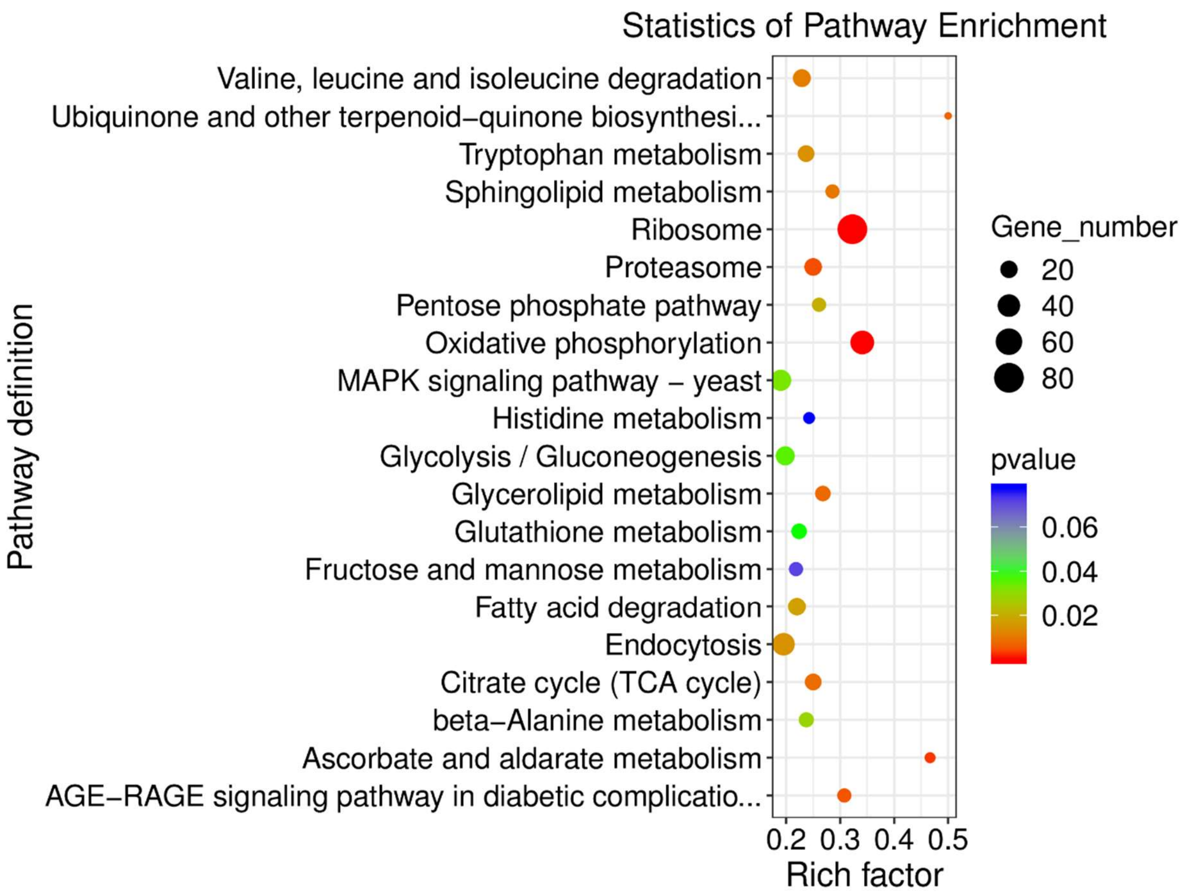

Figure S2. KEGG enriched pathways between supplemented and non-supplemented groups.

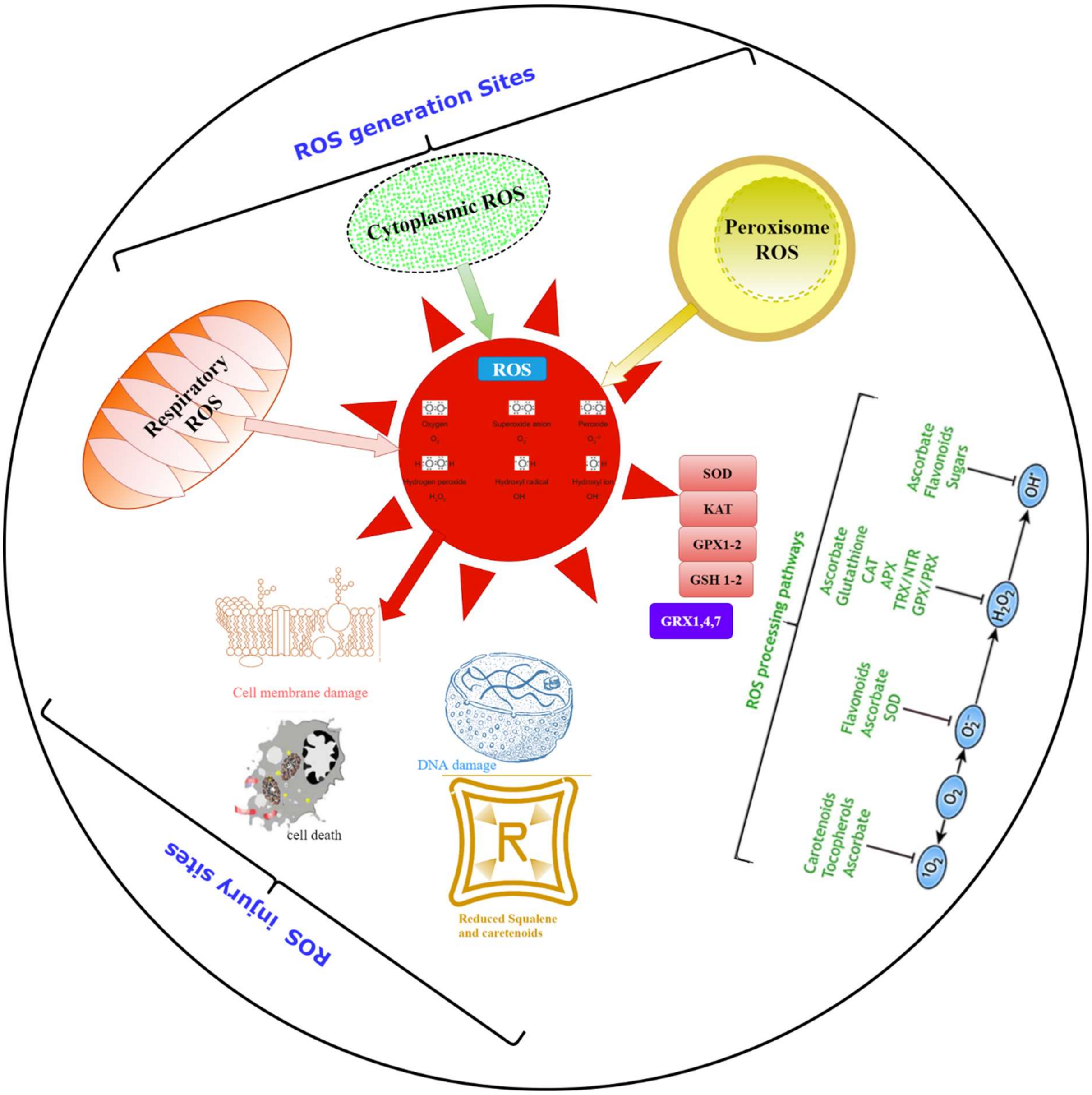

Figure S3. Graphical representation of ROS types, generation sites, damage sites and genes responsible for neutralization of ROS in biological system. Red box shows up regulated genes and blue box shows down regulated genes.
